# Short-Term Effects of *Trans*-Cinnamic Acid on the Metabolism of *Zea mays* L. Roots

**DOI:** 10.3390/plants12010189

**Published:** 2023-01-02

**Authors:** David López-González, Leonardo Bruno, Carla Díaz-Tielas, Antonio Lupini, Meriem Miyassa Aci, Emanuela Talarico, Maria Letizia Madeo, Antonella Muto, Adela M. Sánchez-Moreiras, Fabrizio Araniti

**Affiliations:** 1 Departmento de Bioloxía Vexetal e Ciencia do Solo, Facultade de Bioloxía, Universidade de Vigo, Campus Lagoas-Marcosende s/n, 36310 Vigo, Spain; 2 Dipartimento di Biologia, Ecologia e Scienza della Terra, Università della Calabria (DiBEST-UNICAL), 87036 Arcavacata di Rende, Italy; 3 Dipartimento Agraria, Università Mediterranea di Reggio Calabria, 89124 Reggio Calabria, Italy; 4 Dipartimento di Scienze Agrarie e Ambientali—Produzione, Territorio, Agroenergia, Università Statale di Milano, Via Celoria n°2, 20133 Milano, Italy

**Keywords:** lignin, maize, metabolomics, root, stress, *trans*-cinnamic acid

## Abstract

*trans*-Cinnamic acid is a phenolic compound widely studied in plant metabolism due to its importance in regulating different plant processes. Previous studies on maize plants showed that this compound could affect plant growth and causes metabolic changes in the leaves when applied. However, its effects on root metabolism are not well known. This study analyses the short-term effect of *trans*-cinnamic acid on the morphology of vascular bundle elements and metabolism in maize roots. At short times (between 6 and 12 h), there is a reduction in the content of many amino acids which may be associated with the altered nitrogen uptake observed in earlier work. In addition, the compound caused an alteration of the vascular bundles at 48 h and seemed to have changed the metabolism in roots to favor lignin and galactose synthesis. The results obtained complement those previously carried out on maize plants, demonstrating that in the short term *trans*-cinnamic acid can trigger stress-coping processes in the treated plants.

## 1. Introduction

Allelopathy is a phenomenon whereby specialized metabolites produced by living organisms (bacteria, fungi, viruses or plants) can positively or negatively affect the development of biological or agricultural systems [1]. Those characteristics make this ecological phenomenon a potential tool for the development of new botanical herbicides employable in sustainable weed management [2]. There is a wide variety of specialized metabolites, which can be grouped into three main groups: terpenoids, nitrogen compounds and phenolics [3]. Given the problems with the limited number of modes of action (MOAs) of current herbicides, numerous studies are focusing on the search for new MOAs using these specialized metabolites, which are characterized by a high structural diversity [4]. This high structural diversity makes possible that specialized metabolites of the same class might have different effects on plant metabolism. On the other side, compounds from different classes may have similar effects.

Harmaline, for example, is an indole alkaloid that alters the balance between the phytohormones auxin, cytokinin and ethylene, altering the development of *Arabidopsis thaliana* (L.), Heynh. seedlings [5]. Another indole alkaloid such as norharmane can decrease the growth of *A. thaliana* seedlings by altering the distribution of auxin transporter proteins (PINs) [6], and of adult *A. thaliana* plants by generating water stress when applied by irrigation, while inhibiting the germination and growth of some weed species [7]. Another compound capable of generating water stress in *A. thaliana* adult plants is the monoterpene citral, which also inhibited the development of different weed species [8]. At the same time, farnesene, another terpenoid, was able to alter the distribution of the phytohormone auxin, which led to alterations in the meristem and caused a “left-handedness” phenotype [9].

Phenolic compounds have been studied for many years for their physiological and biochemical effects on plants as they actively participate in the response of plants to abiotic and biotic stress [10,11], particularly for their antioxidant properties [12,13]. Phenolic compounds are important not only for their effects on plants but also for their numerous antimicrobial [14,15], pharmacological [16,17] and industrial [18,19] properties. It is also important to highlight their role in the food industry because of their impact on so-called functional foods, which, in addition to their nutritional value, contain biologically active components with beneficial impact on health, reducing the risk of contracting certain diseases [20,21].

Cinnamic acids are a class of phenolic compounds studied for many years. Whitehead [22] observed how these acids accumulate in the soil after the decomposition of organic matter. These compounds can be presented as *cis-* or *trans*-isomers, and their activity depends on the type of isomer [23]. In particular, *cis*-form is more active than *trans*-form [24]. They are important for plant growth and development as they have protective functions and are key compounds in regulating the phenylpropanoid pathway [25]. However, high concentrations of these acids can trigger plant damage, such as alterations in membrane permeability [26], phytohormonal activity [27], or generation of oxidative stress [28], among others.

Lupini et al. [29] found that exogenous application of *trans*-cinnamic acid to maize plants inhibited plasma membrane H^+^-ATPase activity, which reduced nitrate uptake and caused a reduction in root growth as well. In addition, Araniti et al. [30] showed that the application of *trans*-cinnamic acid to maize leaves causes stress to the plants, but they can cope with this stress by redirecting metabolism towards the production of protective metabolites such as galactose or ascorbic acid. Regarding the effects on roots, Salvador et al. [31] showed that different concentrations of *trans*-cinnamic acid (from 0.1 to 1 mM) increased indole-3-acetic acid (IAA) oxidase and cinnamate 4-hydroxylase (C4H) activities after 24 h of treatment. The increase in C4H activity led to an increase in lignin content which, together with the rise in IAA oxidase activity, led to a decrease in *Glycine max* L. Merr. root growth.

However, no metabolomic studies and short-term effects of *trans*-cinnamic acid have been studied up to now on treated roots, which could give light on the primary effects of *trans*-cinnamic acid on root metabolism.

Metabolomics is a handy tool used to observe changes in the metabolome of plants treated with phytotoxic compounds or to study metabolite changes under stress [32,33,34].

For this reason, a metabolomics approach accompanied by measurements of vascular bundle components was performed to observe the short-term changes that *trans*-cinnamic acid ED_50_ produced on *Zea mays* L. roots.

## 2. Results

### 2.1. Xylem Measurements

**Figure 1:**
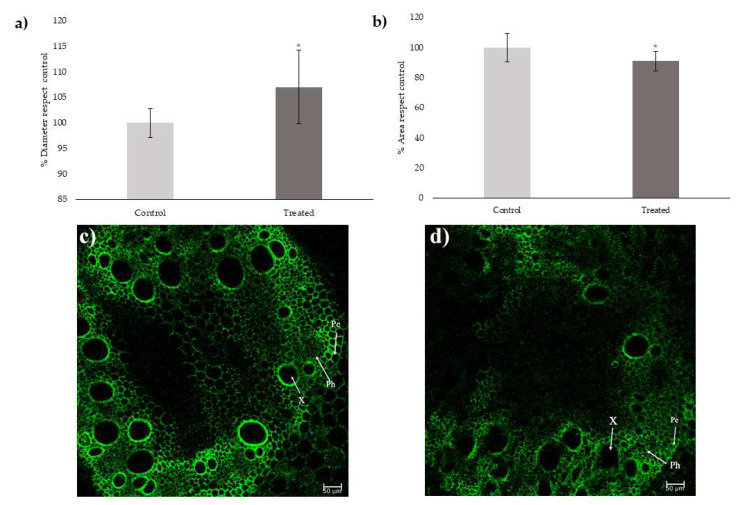
(**a**) Diameter of the vascular bundle after 48 h of treatment with *trans*-cinnamic acid expressed as a percentage with respect to the control. (**b**) Xylematic area after 48 h of treatment with *trans*-cinnamic acid expressed as percentage respect to the control. (**c**) Cross section of a maize control root. (**d**) Cross section of a maize *trans*-cinnamic treated root. (X), xylem; (Ph), phloem; (Pc); pericycle. (*) Indicates significant differences compared to the control (* *p* ≤ 0.05, ** *p* ≤ 0.01, *** *p* ≤ 0.001). Scales bars = 50 μm. Data were analysed with a *t*-test at *p* ≤ 0.05.

Treatment with the ED_50_ of *trans*-cinnamic caused alterations in the treated roots’ measured parameters (Figure 1c,d). The compound caused a significant increase of 7% compared to control in the diameter of the vascular cylinder (Figure 1a), although the area of the xylem vessels was significantly reduced by 9% compared to control in the treated roots (Figure 1b).

### 2.2. Untargeted Metabolomic Analysis

GC-MS-driven untargeted-metabolomic analysis was carried out to get more insights into the metabolic changes produced by the *trans*-cinnamic on maize seedlings at different treatment times, from 6 to 48 h.

A total of 651 compounds were identified using the MS-DIAL software; of these, 360 were unknown, whereas 291 were putatively annotated following the metabolomic society initiative (Supplementary Table S1). After manual feature annotation and discarding false annotated metabolites, a total of 134 primary and specialized metabolites were identified.

Data were analysed through unsupervised multivariate analysis to reduce the dimensionality of the data and visualize how they were grouped. The PCA’s score plot, built by virtue of the two components PC1 vs. PC2, described the 48.3% of the total variability (Figure 1a). PC1 explained the highest variance (27.8%), while PC2 explained the 20.5% of the total variance. The PCA revealed discrimination between the groups, but this separation was unclear.

**Figure 2:**
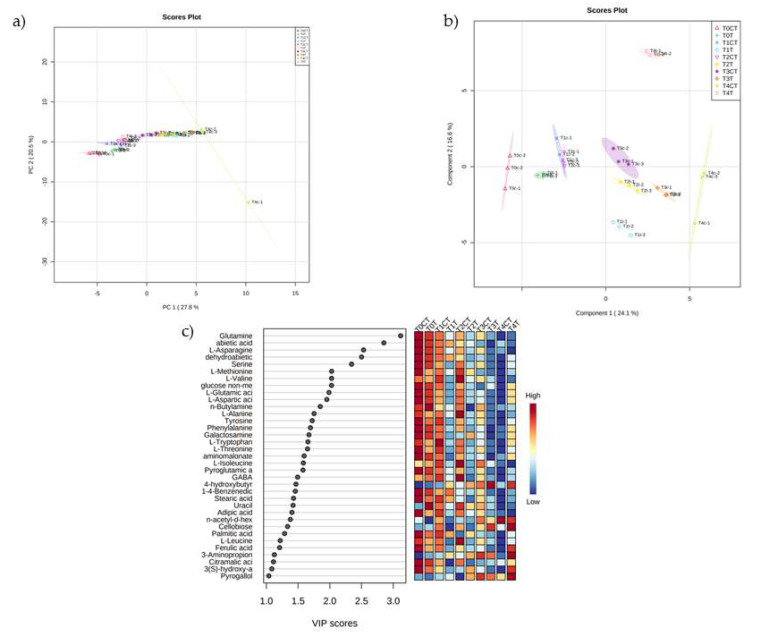
(**a**) Unsupervised PCA of the metabolomic changes on maize roots after 48 h treatment with 103 μM of *trans*−cinnamic acid. (**b**) Multivariate (PLS−DA) analysis of the metabolomic changes on maize roots after 48 h treatment with 103 μM of *trans*−cinnamic acid. (**c**) Important features identified by PLS−DA. The coloured boxes on the right indicate the relative concentrations of the corresponding metabolite in each group under study Times (T0 = 0 h; T1 = 6 h; T2 = 12 h; T3 = 24 h; T4 = 48 h). The treatments used were 0 μM (control, CT), and (treatment, T). N = 3.

Then, the supervised PLS-DA showed the separation between control and treatments at different times (Figure 2b). The model was characterized by a R^2^, Q^2^ and accuracy higher than 0.8 (Supplementary Table S1), indicating good predictivity and high fitting of this model. The separation was achieved by virtue of the first two principal components (PC1 vs. PC2), which explained a total variance of 40.7%. Component 1 explained the highest variance (24.1%), while component 2 explained 16.6% of the total variance. The *trans*-cinnamic treatments, at times 1, 2 and 3, were closer to the control at T3 than their respective controls (T1 and T2). This may indicate a strong short-term acceleration of metabolism by the compound, which is maintained over time. After a long time (T4), there was a clear separation between treatment and control, mainly due to PC2, indicating a significant difference between the metabolomes of control and treated plants.

**Figure 3:**
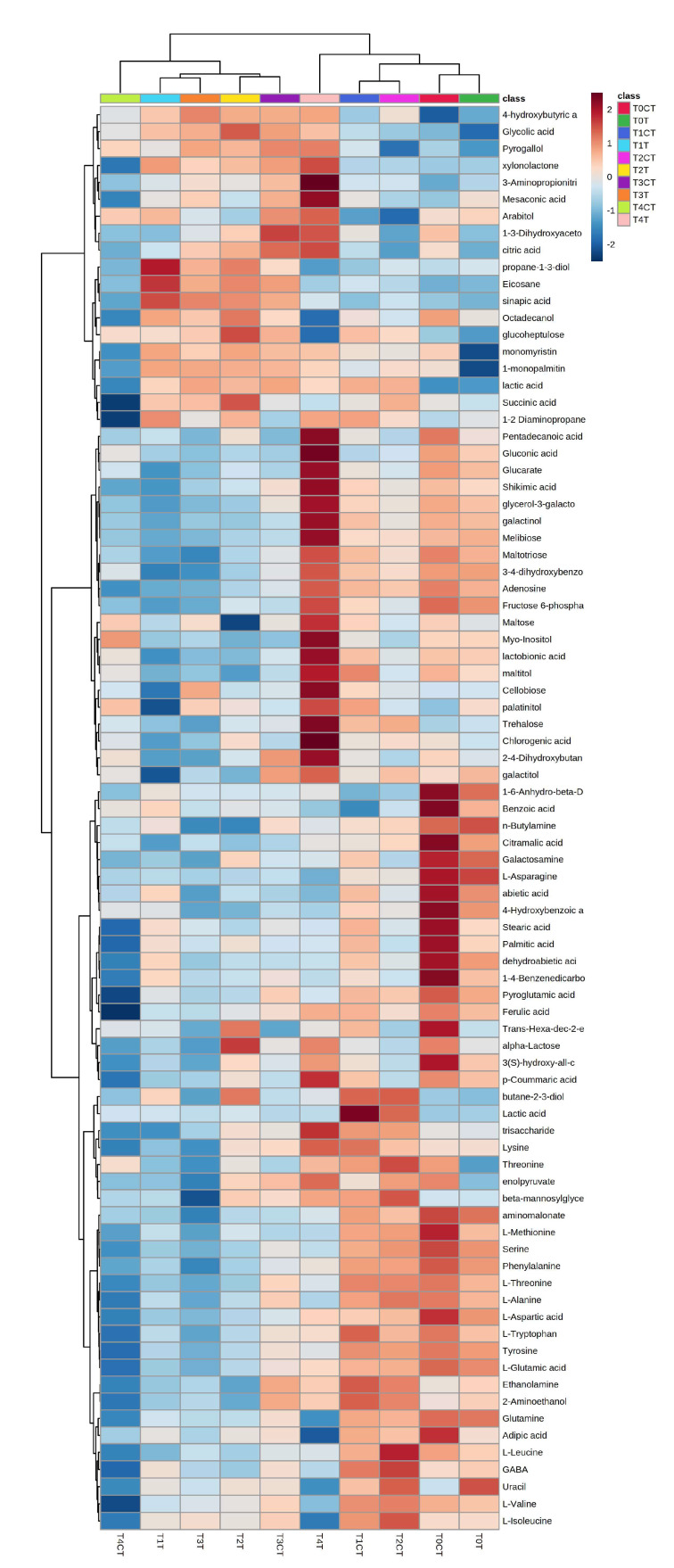
Clustering result shown as heatmap (distance measure using Euclidean, and clustering algorithm using Ward method) of all the metabolites identified in seedlings exposed to trans-cinnamic acid compared to control. Each square represents the effect of *trans*−cinnamic acid on the amount of each metabolite using a false−colour scale. Red or blue regions indicate increased or decreased metabolite content, respectively. Times (T0 = 0 h; T1 = 6 h; T2 = 12 h; T3 = 24 h; T4 = 48 h). The treatments used were 0 μM (control, CT), and 103 μM (treatment, T) N = 3.

PLS-DA derived variable importance of projection (VIP) scores (built on the metabolites with a VIP score higher than 1.4) (Figure 2c) revealed that glutamine, abietic acid, L-asparagine, dehydroabietic acid, serine, L-methionine, L-valine, glucose, L-glutamic acid, L-aspartic acid, n-butylamine, L-alanine, tyrosine, phenylalanine, galactosamine, L-tryptophan, L-threonine, aminomalonate, L-isoleucine, pyroglutamic acid, gamma-aminobutyric acid (GABA), 4-hydroxybutyric acid, 1-4-benzenedicarboxylic acid, stearic acid, uracil, and adipic acid were the metabolites that were mainly contributing to groups discrimination (Figure 3).

A univariate ANOVA was performed to find which metabolites were significantly altered among treatments. The analysis revealed that 84 out of the 134 compounds identified were significantly altered (Supplementary Table S1). Those metabolites were presented on a heatmap showing how each metabolite varied according to time and treatment (Figure 3). The trend observed in the heat map showed the same dynamics as discussed in the PLS-DA. At T1, T2 and T3, there was a general decrease in the accumulation of several amino acids, such as glutamine, glutamic acid, alanine, aspartate, tyrosine, tryptophan, serine, etc., in the roots treated with *trans*-cinnamic acid compared to their controls. While at T4 it could be observed that the *trans*-cinnamic treatment resulted in the accumulation of many of the identified metabolites compared to the control, such as citric acid, lactic acid, shikimic acid, galactinol, melibiose, maltose, myo-inositol, cellobiose, trehalose, phenylalanine, chlorogenic acid, galactitol, ferulic acid, α-lactose, *p*-coumaric acid, or adipic acid among others.

A detailed analysis of the pathways and networks affected by trans-cinnamic treatment was performed using the “MetPa” module of Metaboanalyst. The analysis revealed that 39 routes were significantly affected; 14 had an impact score higher than 0.20 (Supplementary Table S1 and Table 1). In particular, the main pathways affected were: (i) biosynthesis of secondary metabolites—unclassified; (ii) alanine, aspartate and glutamate metabolism; (iii) isoquinoline alkaloid biosynthesis; (iv) phenylalanine metabolism; (v) cyanoamino acid metabolism; (vi) glycine, serine and threonine metabolism; (vii) beta-alanine metabolism; (viii) starch and sucrose metabolism; (ix) tyrosine metabolism; (x) galactose metabolism; (xi) arginine biosynthesis; (xii) cutin, suberine and wax biosynthesis; (xiii) glyoxylate and dicarboxylate metabolism; and (xiv) citrate cycle (TCA cycle).

## 3. Discussion

In previous works with *trans*-cinnamic acid, Lupini et al. [29] showed that this compound had a strong phytotoxic effect on maize seedlings roots after 2 days of treatment, which varied according to the root type (primary, seminal, nodal and laterals). The growth inhibition observed in this study was related to the inhibition of the plasma membrane H^+^-ATPase activity that would negatively alter nitrate uptake and acidification, thereby reducing cell wall loosening. Later, Araniti et al. [30] observed that after 5 days of treatment with *trans*-cinnamic acid on maize leaves, the compound caused stress, reducing leaf growth and seedling development. However, the plants could cope with this stress by producing stress response-related metabolites such as salicylic acid. Moreover, treated plants were redirecting the metabolism to galactose production, leading to an increase in ascorbic acid content, an important compound in plant protection during oxidative stress. In this study, we focused on the short-term (6 to 48 h treatment) effects of *trans*-cinnamic acid on maize seedling roots, focusing on its effect on xylem vessels and how metabolism varied over time.

In cross-sections of maize roots after 48 h of treatment with ED_50_ of *trans*-cinnamic acid, it could be observed how the compound caused an increase in the diameter of the vascular cylinder and a reduction in the xylem vessels’ area. An increase in the diameter of the vascular cylinder could be observed in situations of metal stress, such as cadmium in *Glycine max* L. [35] or under moderate salinity stress in soybean [36]. This alteration under salt stress was also observed in plants growing in arid areas, such as *Tamarix ramosissima* Ledeb. [37]. The increase in the diameter of the vascular cylinder after treatment with *trans*-cinnamic acid might indicate that the plants started to be stressed. Reduced xylem diameter observed after treatment may also be a symptom of stress. Lovisolo and Schubert [38] observed that *Vitis vinifera* L. plants subjected to water stress had a smaller xylem vessel area. Other species of commercial interest, such as tomato (*Solanum lycopersicum* L.) [39] and soybean [40], also saw their xylem area reduced when subjected to water stress. This alteration was also observed in pearl millet (*Pennisetum glaucum* [L.] R. Br.), a cereal species adapted to semi-arid environments [41]. Therefore, the alterations observed in *trans*-cinnamic acid-treated roots, joined to those observed by Lupini et al. [29], suggest that plants are experiencing stress and that their growth and development are compromised. Salvador et al. [31] also demonstrated that hosoybean roots’ growth was altered after cinnamic acid treatment due to increased lignification and auxin alteration after 24 h treatment. However, no studies were previously done at very short times (under 24 h) to understand the primary mechanism of action of *trans*-cinnamic acid. Therefore, metabolomic measurements at different times for 48 h were taken to observe the changes that occurred in the metabolome over time (from 6 to 48 h).

In the PLS-DA, a clear separation between control and treatment was observed at tested times, reflected in the heatmap built using the significantly altered compounds resulting from the ANOVA analysis. It could be observed how the treatment with *trans*-cinnamic acid had already altered the metabolism after 6 h exposure to the compound, and that the variation in the metabolites was similarly maintained up to 24 h of treatment. This early variation was characterized by a decrease in the content of most amino acids as determined by GC-MS. This alteration in the levels of many amino acids could also be observed in the analysis of metabolic pathways, where up to six pathways related to amino acid metabolism were altered (alanine, aspartate and glutamate metabolism; phenylalanine metabolism; glycine, serine and threonine metabolism; beta-alanine metabolism; tyrosine metabolism; and arginine biosynthesis). The plant can take amino acids directly from the medium, but these are generally synthesised from the ammonium or nitrate that the plants absorb from the soil circulating solution [42]. The main site of formation is in the leaves, but amino acid is also synthesized in the roots [43]. Glutamine synthetase and glutamate synthase are the first enzymes involved in the amino acid synthesis, originating from glutamine and glutamate, from which most of the remaining amino acids are produced [44]. Therefore, low levels of these amino acids, such as those observed in the early times (T1, T2 and T3) of treatment with *trans*-cinnamic acid, can cause a drop in the content of the other amino acids. Lupini et al. [29] demonstrated how *trans*-cinnamic acid causes inhibition of nitrogen uptake in maize roots after 24 h of treatment. Several studies have also shown that plants growing on nitrogen-poor media show a low amino acid content [45,46]. Therefore, the inhibition of nitrogen uptake caused by *trans*-cinnamic acid could be the cause behind the decrease in amino acids observed in this study at very short treatment times, which could be considered the primary mechanism of action of the compound. This decrease in amino acids over time would also induce a reduction in protein synthesis, as observed by Araniti et al. [30] in their experiment with *trans*-cinnamic acid.

On the contrary, at 48 h of treatment the variation in metabolites was very different from the rest of the times and the control. As shown in the heatmap, it could be observed that after 48 h treatment many of the metabolites related to lignin syntheses, such as phenylalanine, ferulic acid or p-coumaric acid, were increased compared to the control [47].

Lignin biosynthesis begins with the transformation of phenylalanine into cinnamic acid, which is then transformed into p-coumaric acid [48]. These transformations are the first step in lignin and flavonoid synthesis [49]. The increase in phenylalanine and *p*-coumaric acid suggests an alteration in the phenylpropanoid pathway, confirmed by metabolic pathway analysis, where one of the affected pathways was phenylalanine metabolism. The addition of the compound could trigger metabolic pathways (such as lignin biosynthesis) to reduce the excess of *trans*-cinnamic acid and detoxify it from the cells. D’Apice et al. [50] observed that alterations in phenylalanine metabolism led to increased lignin synthesis and changes in many metabolites involved in this process.

Lignin is a biopolymer normally made up of three basic units of natural lignin polymers: p--hydroxyphenyl (H), guaiacyl (G), and syringyl (S), which are generated from three monolignols, p--coumaryl alcohols, coniferyl alcohols, and sinapyl alcohols, respectively [49]. A metabolite also involved in lignin synthesis, specifically in S-subunits, is sinapic acid [49]. Although this metabolite was decreased after 48 h of treatment, its levels were elevated until 24 h of treatment with *trans*-cinnamic acid, as seen in the heat map, suggesting that this compound was consumed over time to increase the synthesis of S-subunits.

Other compounds that accumulated in the roots after 48 h of treatment with the compound were shikimic acid and chlorogenic acid, which also influence lignin biosynthesis [51] and trehalose. Trehalose is synthesised from glucose-6-phosphate and uracil (another accumulated metabolite) and can activate lignin biosynthesis [52].

Increased lignin synthesis can improve resistance to lodging [53] or to biotic or abiotic stress [49], but it can also lead to growth inhibition, as observed by Deng et al. [54] after treating tomato plants with the natural growth regulator laxogenin C. The reduction in root growth in maize plants observed by Lupini et al. [29] after treatment with *trans*-cinnamic acid could be a consequence of an increase in lignification caused by an alteration in phenylalanine metabolism.

Finally, Araniti et al. [30] observed how galactose metabolism was affected in maize leaves in response to *trans*-cinnamic acid stress after 5 days. The accumulation of melibiose, galactinol, maltose, myo-inositol and α-lactose suggests that galactose metabolism may be affected, confirmed by analysis of metabolic pathways. Therefore, the mechanisms against the stress generated by *trans*-cinnamic early on amino acids after 6 h of treatment evolve to lignin production at about 48 h of treatment.

## 4. Conclusions

The results obtained in this study support those obtained by Lupini et al. [29] and Araniti et al. [30] in previous works with *trans*-cinnamic acid, in addition to providing new information on changes in the metabolome of maize roots treated with the compound. At very short times (from 6 to 24 h), the compound causes a decrease in many amino acids and alteration of many related metabolic pathways, confirming that inhibition of nitrogen uptake would be a primary mechanism of action of *trans*-cinnamic acid. In the last tested time (48 h), treatment with *trans*-cinnamic acid caused alterations in both root morphology (changes in the vascular cylinder) and metabolism. In an attempt to detoxify the excess of *trans*-cinnamic acid, the plant appears to convert it into lignin by activating phenylalanine metabolism. The compound also seems to increase galactose synthesis to cope with the stress generated by *trans*-cinnamic acid. Finally, it will be interesting and meaningful to investigate by molecular approaches based on NGS (RNA sequencing or ChipSeq) to provide useful information regarding the molecular mechanisms involved in plant response to *trans*-cinnamic acid.

## 5. Materials and Methods

### 5.1. Plant Material, Growth Conditions and Treatment

Seeds of maize (*Zea mays* L.) were primed and germinated according to [29]. After germination, plants with uniform size were transferred in hydroponic systems with a one-fourth strength Hoagland solution [29]. Seedlings were maintained in this solution for 48 h under the conditions described by [30]. Then seedlings were transferred for 2 days to the same medium with 103 μM *trans*-cinnamic acid. This value is the ED_50_ reported by [29] in previous studies. For the metabolomic assay, plants were harvested at different times (T0 = 0 h; T1 = 6 h; T2 = 12 h; T3 = 24 h; T4 = 48 h). The treatment and solution were renewed daily to maintain nutrients and trans-cinnamic concentrations constant and to avoid the compound’s possible transformation and/or degradation.

### 5.2. Measurement of Xylem Area

After 48 h of treatment treated and untreated seedlings were collected and immediately processed. Roots’ cross sections, thick 50 µm, were cut by a vibrotome (Leica VT1000E, Leica Biosystems 21440 W. Lake Cook Road Floor 5 Deer Park, IL 60010 United States) and subsequently mounted on microscope slides and observed under Leica inverted TCS SP8 confocal scanning laser microscope equipped with 20× and 40×/oil immersion objectives. Argon laser excitation wavelength at 488 nm and an emission window of 509 nm were used. The diameter of the vascular bundle and the area of the xylem were evaluated through the image processing and analysis program ImageJ (http://imagej.nih.gov/ij/docs/index.html; accessed on 1 September 2022). Both parameters were calculated as % compared to the control.

### 5.3. Untargeted Metabolomic Analysis

The effect of *trans*-cinnamic acid on the metabolism of *Zea mays* plants was evaluated at different treatment times (T0 = 0 h; T1 = 6 h; T2 = 12 h; T3 = 24 h; T4 = 48 h). The treatments used were 0 μM (control, CT), and 103 μM (treatment, T) of *trans*-cinnamic acid. Roots were collected at different times and frozen in liquid nitrogen to stop metabolism. Subsequently, samples were ground and 100 mg of plant material per replicate were weighed and placed in 2 mL vials.

For the extraction, 1400 μL of methanol (−20 °C) were added to the plant material and shaken for 10 s. As a quantitative internal standard, 60 μL of ribitol (0.2 mg mL^−1^ stock in ultrapure H_2_O) were added. Samples were placed in a thermomixer at 70 °C under agitation for 10 min (950 rpm) and then centrifuged for 10 min at 11,000× *g*. The supernatant obtained was transferred to glass vials where 750 µL CHCl_3_ (−20 °C) and 1500 µL ultrapure H_2_O (4 °C) were sequentially added to carry out the separation of metabolites by their polarity. Vials were vortexed for 10 s and then centrifuged for 15 min at 2200 g. After centrifugation, 150 µL of the upper phase (polar phase) were taken and placed in 2 mL vials to be completely dried in a vacuum concentrator without heating. The samples’ derivatisation was carried out in two steps. The first step was the metoximation of the samples, which was achieved by adding 40 µL of methoxyamine hydrochloride (20 mg mL^−1^ in pyridine) to the dried samples and incubating them for 2 h in a thermomixer at 37 °C (950 rpm). The second step of the derivatization consisted of the silylation of the samples, achieved by adding 70 µL of MSTFA to the aliquots. Samples were then shaken in a thermomixer at 37 °C (950 rpm) per 30 min. Finally, 110 µL of the derivatised samples were transferred into glass vials for GC-MS analysis.

### 5.4. GC-Quadrupole/MS Analysis

The derivatised extracts were injected into a MEGA-5MS capillary column (30 m × 0.25 mm × 0.25 µm equipped with 10 m of pre-column) using a gas chromatograph apparatus (Agilent 7890A GC, Cernusco sul Naviglio, Milan, Italy) equipped with a single quadrupole mass spectrometer (Agilent 5975C, Cernusco sul Naviglio, Milan, Italy). The injector temperature was set at 250 °C, and the source temperature was set at 260 °C. One µL of the sample was injected in splitless mode with helium as a gas carrier (flow of 1 mL min^−1^) using the following programmed temperature: isothermal 5 min at 70 °C followed by a 5 °C/min ramp to 350 °C and a final 5 min heating at 330 °C. Mass spectra were recorded in electronic impact (EI) mode at 70 eV, scanning at 40–600 m/z range, scan time 0.2 sec. Mass spectrometric solvent delay was settled as 9 min. Blank solvents (pyridine), n-alkane standards and pooled samples that served as quality control (QCs), were injected at scheduled intervals for instrumental performance, tentative identification, and monitoring of shifts in retention indices (RI). Solvent blanks were run between samples, and each mass was checked against the blank run to exclude possible contamination sources.

### 5.5. Analysis of GS-MS Data by MS-DIAL

The MS-DIAL software, with an open-source publicly available EI spectra library, was used for raw peaks extraction, data baseline filtering and calibration of the baseline, peak alignment, deconvolution analysis, integration of the peak height and peak annotation. The average peak width of 20 scans and a minimum peak height of 1000 amplitudes was applied for peak detection, and the sigma window value of 0.5, EI spectra cut-off of 10 amplitudes was implemented for deconvolution. For peaks identification, the retention time tolerance was settled at 0.5 min, the m/z tolerance was 0.5 Da, the EI similarity cut-off was 70%, and the identification score cut-off was 70%. The alignment parameters setting process and the retention time tolerance was 0.075 min.

We used publicly available libraries for compound annotation based on the mass spectral pattern as compared to EI spectral libraries such as the MSRI spectral libraries from Golm Metabolome Database [55] available from Max-Planck-Institute for Plant Physiology (Golm, Germany) and MassBank [56], MoNA (Mass Bank of North America).

Once the compounds and features were identified and annotated, the shared metabolites were only reported as quantified and confidently identified. For metabolite annotation and assignment of the EI-MS spectra, we followed the metabolomics standards initiative (MSI) guidelines for metabolite identification [57]. In particular, samples were annotated at: (i) Level 2: identification was based on the spectral database (match factor >70%); and (ii) Level 3: only compound groups were known, e.g., specific ions and RT regions of metabolites.

### 5.6. Statistical Analysis

GC-MS-driven untargeted metabolomics were carried out using a randomised design with three replications (*n* = 3).

Metabolomic data were normalised using the internal standard and analysed using Metaboanalyst 5.0 [58]. The missing values of the Lowess normalised dataset were replaced with a half of the minimum value found in the data set. Successively, data were Log_10_ transformed, and Pareto scaled. Data were then classified through Principal Component Analysis (PCA). PLS-DA was also employed to identify the differential metabolites by calculating the corresponding variable importance in the projection (VIP value). Finally, data were also analysed through the univariate analysis one-way ANOVA using the LSD test as post-hoc (*p* ≤ 0.05). A false discovery rate was applied to the nominal *p*-value as a control for false-positive findings. Metabolites significantly affected by the treatments in the ANOVA test were presented as a heatmap and clustered using the Euclidean distance measurement and the Ward method for groups clusterisation.

Pathways analysis was carried out using the Metaboanalyst 5.0 tools and setting *Oryza sativa* L. as a metabolome reference database.

## Figures and Tables

**Table 1:** Results from ingenuity pathway analysis with MetPa carried out on *Zea mays* roots treated with 103 μM *trans*-cinnamic acid after exposure to various times with the compound.

Pathway	Total Cmpd	Hits	Impact	Raw *p*	FDR
Biosynthesis of secondary metabolites—unclassified	5	1	1	0.00076429	0.0014331
Alanine, aspartate and glutamate metabolism	22	9	0.77698	2.88 × 10^−5^	0.00045239
Isoquinoline alkaloid biosynthesis	6	2	0.64705	0.00052097	0.0010779
Phenylalanine metabolism	12	1	0.42308	0.0035621	0.0048574
Cyanoamino acid metabolism	26	4	0.375	0.0013535	0.0021949
Glycine, serine and threonine metabolism	33	6	0.3547	0.00048176	0.0010323
beta-Alanine metabolism	18	4	0.3254	1.20 × 10^−5^	0.00045239
Starch and sucrose metabolism	22	5	0.32054	0.0016991	0.0025486
Tyrosine metabolism	18	4	0.27568	0.0014806	0.0023377
Galactose metabolism	27	8	0.26927	0.0009734	0.0016687
Arginine biosynthesis	18	6	0.25243	0.00011212	0.00051746
Cutin, suberine and wax biosynthesis	14	2	0.25	0.00071144	0.001377
Glyoxylate and dicarboxylate metabolism	29	7	0.23322	6.03 × 10^−5^	0.00045239
Citrate cycle (TCA cycle)	20	5	0.23269	0.019317	0.022726

Total Cmpd: the total number of compounds in the pathway; Hits: is the matched number from the uploaded data; Impact: is the pathway impact value calculated from pathway topology analysis; Raw *p*: is the original *p* value calculated from the enrichment analysis; FDR: false discovery rate; N = 3.

## Data Availability

The datasets generated during and/or analysed during the current study are available from the corresponding author on reasonable request.

## Supplementary Materials

The following supporting information can be downloaded at: https://www.mdpi.com/article/10.3390/plants12010189/s1, Table S1: untargeted metabolomics raw and statistical data.
